# Mortality and complications after hip fracture among elderly patients undergoing hemodialysis

**DOI:** 10.1186/s12882-015-0099-0

**Published:** 2015-07-07

**Authors:** Jeff Chien-Fu Lin, Wen-Miin Liang

**Affiliations:** Department of Statistics, National Taipei University, Taipei, Taiwan; Department of Orthopedic Surgery, Wan Fang Hospital, Taipei Medical University, Taipei, Taiwan; Graduate Institute of Biostatistics, Biostatistics Center, Department of Public Health, China Medical University, Taichung, Taiwan

**Keywords:** End-stage renal disease, Hemodialysis, Osteoporotic hip fracture, Mortality, Surgical complication, Medical complication, Elderly patient

## Abstract

**Background:**

Osteoporotic hip fractures cause high mortality and morbidity in elderly adults. Compared to the general population, subjects with end-stage renal disease and hemodialysis often develop mineral bone disorders and have a higher risk for hip fractures.

**Methods:**

We conducted a matched cohort study design and used competing risk analysis to estimate the cumulative incidence of the complication rate. Subjects aged greater than 60 years with hip fracture were selected from Taiwan’s National Health Insurance Research Database covering a period from 1997 to 2007, and these subjects were followed up until 2009. We used the Kaplan–Meier method to estimate the overall survival and used the log-rank test and multiple Cox proportional hazards model to explore the risk factors for survival. The cumulative incidence of the first complication was estimated using competing risk analysis.

**Results:**

Among hemodialysis subjects, the three-month, one-year, two-year and five-year mortality rates were 17.3 %, 37.2 %, 51.5 %, and 80.5 %, respectively; the one-year and five-year cumulative incidences of the first surgical complication were 14.2 % and 20.6 %, respectively; and the three-month cumulative incidence of the first medical complication was 24.1 %. Hemodialysis subjects presented a 2.32 times (95 % CI: 2.16–2.49) higher hazard ratio of overall death, 1.15 times (95 % CI: 1.01–1.30) higher sub-hazard ratio (sub-HR) of surgical complications, and 1.35 times (95 % CI: 1.21–1.52) higher sub-HR of the first medical complication than non-hemodialysis controls.

**Conclusions:**

The overall mortality and complication rates of hemodialysis subjects after surgery for hip fracture were significantly higher than those of non-hemodialysis subjects. Further prospective studies which include important risk factors are necessary to more precisely quantify the adjusted effect of hemodialysis.

**Electronic supplementary material:**

The online version of this article (doi:10.1186/s12882-015-0099-0) contains supplementary material, which is available to authorized users.

## Background

Osteoporotic hip fractures cause high mortality and morbidity in elderly adults [1–4]. One-year mortality rates after hip fracture were up to 30 % in the elderly [1–4]. After surgery for hip fractures in elderly patients, the short-term readmission or reoperation rates were 5 % to 30 % within 12 months, and the long-term reoperation rates were 20 % to 40 % [5–14].

Compared to the general population, subjects with end-stage renal disease (ESRD) and dialysis often develop mineral bone disorders and had a higher risk for hip fractures [15–23]. Studies reported that one-year mortality rates after hip fracture were up to 30 % and 64 %, with short-term readmission rates up to 40 % in hemodialysis subjects [19, 23–30]. However, few previous studies have reported long-term mortality, surgical and medical complications simultaneously using a large sample size. Therefore, in this study, the short-term and long-term mortalities and complications after surgery for hip fracture were investigated using competing risk analysis in hemodialysis subjects aged greater than 60 years from a nationwide population database in Taiwan.

## Methods

### Data source and subjects

Data were obtained from the National Health Insurance Research Database (NHIRD) released by the National Health Research Institutes in Taiwan. Taiwan began its National Health Insurance program in 1995 to finance health care for all residents. The coverage rate was more than 99 % of the total population of about 23 million residents in 2012. The database includes comprehensive information on insured subjects, such as demographic data, dates of clinical visits, diagnostic codes, details of prescriptions, and expenditure amounts. This study was approved by the Institutional Review Board of China Medical University Hospital.

This study selected subjects aged 60 years old and above, who were admitted to hospitals from January 1997 to December 2007. Subjects were identified both with (i) a first discharge diagnosis code of hip fracture (based on International Classification of Diseases, Ninth Revision, Clinical Modification (ICD-9-CM) codes 820, 820.0, 820.00, 820.01, 820.02, 820.09, 820.8, 820.03, 820.2, 820.20, and 820.21) and (ii) medical code with surgery of internal fixation or hemiarthroplasty (based on ICD-9-CM codes 79.15, 79.35, and 81.52). The first admission date of hip fracture was defined as the index day of surgery. The exclusion criteria were inpatients with pathological fractures (ICD-9-CM codes 733.14 and 733.15) or open hip fractures (ICD-9-CM codes 820.1, 820.10, 820.11, 820.12, 820.19, and 820.9). Subjects who underwent operations on their pelvis, femur, or hip region before the index day were also excluded to avoid confounding effects.

Owing to certain covariate differences, we used a matched cohort design to explore the outcomes of hemodialysis and non-hemodialysis subjects after hip fractures. For each hemodialysis subject, we randomly matched one non-hemodialysis subject, who had the same age, gender, fracture type, operation type, and comorbidities, including hypertension, diabetes, chronic heart disease, and chronic pulmonary disease, to serve as a control. In addition, the non-hemodialysis control had the operation in the same calendar year as the hemodialysis subject. Subjects with chronic renal disease or ESRD without hemodialysis were excluded. In total, 2680 subjects received hemodialysis before the index day of surgery for hip fracture and 2680 matched non-hemodialysis controls were identified. This cohort was followed until death, exiting the NHI program or the end of 2009.

### Ethical approval

All patients’ data were encrypted using the same encryption algorithm to cross-link the data while protecting the privacy of the patients. This study protocol was approved by the Institutional Review Board (IRB) of China Medical University Hospital (protocol # CMUH102-REC2-012).

### Outcomes of interest

This study analyzed three outcomes: (a) overall mortality; (b) cumulative incidence of the first medical complication within 90 days after the index day; and (c) cumulative incidence of the first surgical complication after the index day of surgery. Overall survival time was defined as the duration from the index day to the death day. Subjects who survived at the end of the study or were lost to follow-up were treated as censored. The first surgical complication time was defined as the duration from the index day to the day of the first postoperative unplanned reoperation caused by surgery-related complications. These included converting to arthroplasty or revision arthroplasty, implant failure, surgical site infection, mechanical complications (e.g., loss reduction, screw loosening or cutting out, skin irritation, implant broken/failure), dislocation, avascular necrosis of femoral head, second hip fracture at the same site, and malunion/nonunion during follow-up. The first medical complication time was defined as the duration from the index day to the day of the first medical complication within 90 days after the index surgery, which required extra days of hospital stay or hospital readmission for treatment. The medical complications included stroke, acute myocardial infarction, pulmonary embolism, deep vein thrombosis, acute renal failure, acute respiratory failure, pneumonia, and acute exacerbation of chronic obstructive pulmonary disease (COPD). The comorbidities of a subject were retrieved before or at the time of the index day and included hypertension, diabetes, chronic heart disease, chronic pulmonary disease, cerebrovascular disease, chronic liver disease, and cancer.

### Statistical analysis

We estimated overall survival using the Kaplan–Meier method and explored the risk factors for survival using the log-rank test and the multiple Cox proportional hazards model. We estimated the cumulative incidence of the first complication using competing risk analysis, in which death was considered a competing risk [31–34]. We explored the effects of risk factors on complication-free time using the Gray’s test as well as the Fine and Gray’s model with proportional subdistribution hazards. The risk factors included age, gender, fracture type, operation type, and comorbidities. All analyses were performed using the SAS System (version 9.3; SAS Institute, Cary, NC) and R 3.0.0 [R Development Core Team (2013), R: A language and environment for statistical computing. R Foundation for Statistical Computing, Vienna, Austria, the R libraries survival, cmprsk, and mstate] [35].

## Results

**Fig. 1:**
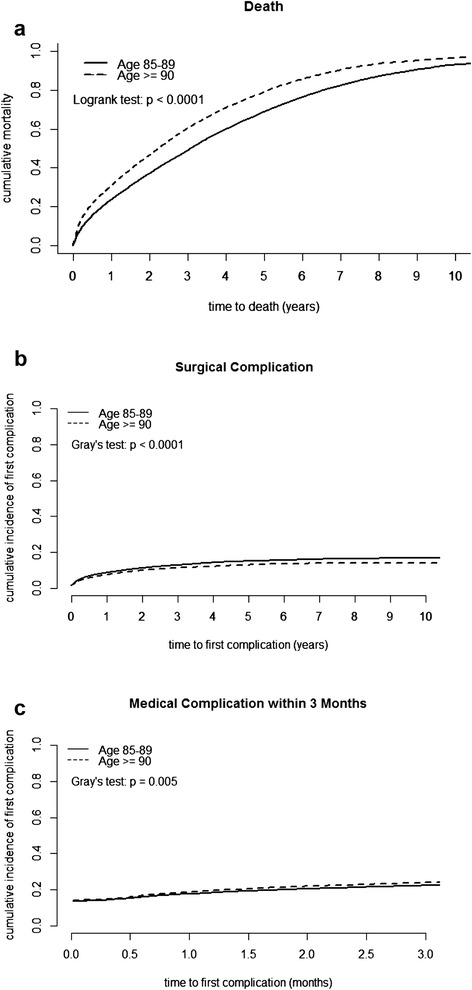
Ten-year cumulative curves of (**a**) mortality stratified by age, (**b**) first surgical complication stratified by age, (**c**) three-month cumulative curves of first medical complication stratified by age

Between 1997 and 2007, 2680 hip fracture subjects with hemodialysis and 2680 matched controls without hemodialysis were identified, of which 63.4 % were female, 36.6 % were male, 56.8 % had cervical fractures, 43.2 % had trochanteric fracture, 53.3 % received internal fixation, and 46.7 % received hemiarthroplasty (Table 1). The death incidence rate was 354.30 per 1000 person-year (PY) (95 % CI: 339.49–369.75) for hemodialysis patients, and 152.04 per 1000 PY (95 % CI: 144.52–159.95) for non-hemodialysis patients. The median survival time was 1.89 years (95 % CI: 1.76–2.03) for hemodialysis, and 4.68 years (95 % CI: 4.42–4.98) for non-hemodialysis subjects. The one-month to ten-year mortality rates and cumulative incidence rates of the first complication are shown in Table 2 and Fig. 1. The two-year and five-year mortality rates were 51.5 % and 80.5 % for hemodialysis and 25.9 % and 52.3 % for non-hemodialysis subjects, respectively. Hemodialysis subjects after surgery for hip fracture had significantly higher overall mortality and complication rates than non-hemodialysis subjects after surgery for hip fracture.

**Table 1:** Baseline characteristics of hip fracture subjects stratified by hemodialysis groups

		Non-hemodialysis (N = 2680)		Hemodialysis (N = 2680)		*p*-value
		N	%	N	%	
Age (years), mean ± SD		74.90 ± 7.04		74.88 ± 7.05		0.912
Gender	Female	1700	(63.4 %)	1700	(63.4 %)	0.999
	Male	980	(36.6 %)	980	(36.6 %)	
Fracture type	Cervical	1521	(56.8 %)	1521	(56.8 %)	0.999
	Trochanteric	1159	(43.2 %)	1159	(43.2 %)	
Operation type	Fixation	1429	(53.3 %)	1429	(53.3 %)	0.999
	Hemiarthroplasty	1251	(46.7 %)	1251	(46.7 %)	
Hypertension	No	651	(24.3 %)	651	(24.3 %)	0.999
	Yes	2029	(75.7 %)	2029	(75.7 %)	
Diabetes mellitus	No	1536	(57.3 %)	1536	(57.3 %)	0.999
	Yes	1144	(42.7 %)	1144	(42.7 %)	
Chronic heart disease^a^	No	1665	(62.1 %)	1665	(62.1 %)	0.999
	Yes	1015	(37.9 %)	1015	(37.9 %)	
Chronic pulmonary disease	No	2254	(84.1 %)	2254	(84.1 %)	0.999
	Yes	426	(15.9 %)	426	(15.9 %)	
Cerebrovascular	No	1506	(56.2 %)	1999	(74.6 %)	<0.001
	Yes	1174	(43.8 %)	681	(25.4 %)	
Chronic liver disease	No	2437	(90.9 %)	2412	(90.0 %)	0.264
	Yes	243	(9.1 %)	268	(10.0 %)	
Cancer	No	2383	(88.9 %)	2387	(89.1 %)	0.896
	Yes	297	(11.1 %)	293	(10.9 %)	

^a^Chronic heart diseases included myocardial infarction, coronary heart disease, congestive heart failure, and arrhythmia

**Table 2:** Cumulative mortality rates and cumulative incidence of first surgical or medical complication after surgery for hip fracture stratified by hemodialysis groups

			Cumulative incidence			
	Mortality		Surgical complication		Medical complication	
Time	Non-HD	HD	Non-HD	HD	Non-HD	HD
1-month	2.1 %	6.9 %	3.7 %	6.0 %	14.7 %	17.7 %
3-month	5.3 %	17.3 %	6.2 %	9.5 %	18.4 %	24.1 %
6-month	9.6 %	26.0 %	8.7 %	11.7 %		
1-year	16.6 %	37.2 %	11.0 %	14.2 %		
2-year	25.9 %	51.5 %	13.5 %	17.1 %		
5-year	52.3 %	80.5 %	18.3 %	20.6 %		
10-year	79.0 %	96.3 %	21.4 %	22.0 %		

*Abbreviations:* *HD* Hemodialysis with hip fracture, *Non-HD* Non-hemodialysis with hip fracture

We found that dialysis, older age, male, trochanteric fracture, hemiarthroplasty, and comorbidities such as diabetes mellitus, chronic heart disease, chronic liver disease, and cancer were significant risk factors for mortality. After controlling for other risk factors, hemodialysis subjects had a 2.32 times (95 % CI: 2.16–2.49) higher hazard ratio (HR) of overall death than non-hemodialysis subjects. We found that dialysis, younger age, cervical fracture, and fixation were significant risk factors for the first surgical complication using multivariable competing risk analysis. Hemodialysis subjects had a 1.15 times (95 % CI: 1.01–1.30) higher sub-hazard ratio (sub-HR) of the first surgical complication than non-hemodialysis subjects. We also found that dialysis, older age, male, trochanteric fracture, hemiarthroplasty, and comorbidities, such as chronic heart disease, chronic pulmonary disease, and cerebrovascular disease, were significant risk factors for the first medical complication. Hemodialysis subjects had a 1.35 times (95 % CI: 1.21–1.52) higher sub-HR of the first medical complication than non-hemodialysis subjects (Table 3).

**Table 3:** (a) Hazard ratios of the risk factors associated with death from cause-specific hazard model (HR) based on Cox model, (b) sub-distribution hazard ratio of the risk factors associated with first surgical complication, and (c) medical complication based on Fine and Gray’s (sub-distribution hazard, sub-HR) model from competing risk analysis

	(a) mortality			(b) surgical complication			(c) medical complication		
	HR	95 % CI	*p*-value	Sub-HR	95 % CI	*p*-value	Sub-HR	95 % CI	*p*-value
Hemodialysis: yes/no	2.319	(2.164–2.485)	<0.001	1.148	(1.013–1.301)	0.031	1.353	(1.208–1.516)	<0.001
Age (1 year)	1.033	(1.028–1.038)	<0.001	0.982	(0.973–0.991)	<0.001	1.040	(1.032–1.049)	<0.001
Gender: male/female	1.279	(1.195–1.369)	<0.001	0.891	(0.782–1.015)	0.082	1.326	(1.186–1.484)	<0.001
Fracture type: trochanteric/cervical	1.235	(1.106–1.380)	<0.001	0.629	(0.517–0.764)	<0.001	1.262	(1.040–1.533)	0.018
Operation type: hemiarthroplasty/fixation	1.182	(1.058–1.319)	0.003	0.827	(0.686–0.996)	0.046	1.219	(1.003–1.480)	0.046
Hypertension: yes/no	0.918	(0.846–0.995)	0.038	0.812	(0.701–0.943)	0.006	0.837	(0.73–0.959)	0.011
Diabetes mellitus: yes/no	1.213	(1.129–1.303)	<0.001	0.917	(0.801–1.051)	0.212	1.018	(0.902–1.15)	0.768
Chronic heart disease^a^: yes/no	1.253	(1.168–1.343)	<0.001	0.898	(0.785–1.027)	0.115	1.138	(1.013–1.279)	0.031
Chronic pulmonary disease: yes/no	1.077	(0.985–1.178)	0.105	0.995	(0.832–1.19)	0.958	2.774	(2.46–3.128)	<0.001
Cerebrovascular disease: yes/no	1.010	(0.939–1.087)	0.783	1.019	(0.889–1.168)	0.792	1.178	(1.045–1.328)	0.007
Chronic liver disease: yes/no	1.193	(1.067–1.333)	0.002	1.023	(0.829–1.263)	0.831	0.891	(0.734–1.083)	0.248
Cancer: yes/no	1.449	(1.313–1.598)	<0.001	0.877	(0.716–1.073)	0.202	0.986	(0.834–1.166)	0.868

^a^Chronic heart diseases included myocardial infarction, coronary heart disease, congestive heart failure, and arrhythmia

The majority of 90-day medical complications were pneumonia (44.89 %), acute respiration failure (40.25 %), acute renal failure pulmonary (23.22 %), exacerbation of chronic obstructive pulmonary disease (18.27 %), and stroke (10.99 %) among 646 hemodialysis subjects with at least one readmission for a medical complication within 90 days after surgery (Additional file 1). Among 460 hemodialysis subjects with at least one reoperation within two years after surgery, the majority of reoperations were due to mechanical complications (33.48 %) and infection (30.87 %). Surgical complications resulted in conversions to arthroplasty or revision arthroplasty (34.35 %) and removal of implants (23.04 %) among these 460 subjects (Additional file 2). Once subjects experienced their first medical complication, 63.6 % of hemodialysis subjects died within one year compared to 36.6 % of the controls. In addition, once subjects had their first reoperation, 41.9 % of hemodialysis subjects died within one year compared to 21.2 % of the controls (Additional file 3).

## Discussion

In this population study, short-term and long-term mortalities and complications after surgery for hip fracture among elderly adults undergoing hemodialysis were analyzed. We found that hemodialysis subjects had significantly higher mortality and complication rates compared to the controls. Hip fracture is one of the most common occurrences in hemodialysis subjects, and hemodialysis subjects had a higher mortality caused by complications of both end-stage renal disease and hip fracture [15–19, 22, 23]. Previous studies reported that one-year mortality rates after hip fracture were up to 30 % and 50 % in hemodialysis subjects [18, 19, 23–30]. We found that the death incidence rate was 354.3 per 1000 PY. The median survival time was 1.89 years, with a one-year mortality rate of 37.2 % for hemodialysis subjects, which fell in the middle of the range reported in previous studies [18, 19, 23–30]. Certain large-scale studies found that hemodialysis subjects not only had a higher mortality rate but also had a higher short-term rehospitalization rate after hip fracture in hemodialysis subjects [23–25]. These studies also reported that comorbidities and medical complications increased mortality and complication after surgery [23–25]. Beaubrun et al. reported that the one-year mortality rate was 630 per 1000 PY and that the one-year rehospitalization rate was 356 per 1000 PY, out of 35,956 hip fractures in more than 200,000 hemodialysis subjects from the US Renal Data System [23]. Mittalhenkle et al. reported a one-year mortality rate of approximately 50 % after hip fracture among 7636 hemodialysis patients from the same database [24]. Mittalhenkle et al. found that the median survival time was 289 days after hip fracture compared to 715 days among hemodialysis subjects without hip fracture based on a matched control study design [24]. Coco and Rush reported that the one-year mortality rate was 64 % for 59 hip fractures in 1272 hemodialysis subjects [19]. Tentori et al. estimated that the one-year mortality rates were more than 500 per 1000 PY among 419 hip fractures in a cohort of 34,579 hemodialysis subjects [25]. Several studies with a small sample sizes reported long-term surgical outcomes after surgery for hip fracture [26–30]. The one-year mortality rates reported in these studies varied widely due to the small sample sizes, with rates of 23 %–50 % [26–30].

We found that hemodialysis subjects had a 2.32 times (95 % CI: 2.17 to 2.49) higher hazard ratio (HR) of overall death than non-hemodialysis subjects. Coco et al., using a similar matched design, showed that the standardized mortality rate of hip fracture for the hemodialysis subjects was 2.4 times greater than that of the general population and that lower parathyroid hormone levels were associated with a higher mortality rate [19]. The very high risk of death after hip fracture in hemodialysis subjects has been shown to be multifactorial. Studies found that age, decline in mobility, malnutrition, decreased muscle strength, lower bone mineral density, abnormal vitamin D metabolism, abnormal parathyroid hormone level, cardiovascular disease, and pneumonia contributed to mortality after hospitalization in hemodialysis subjects [15, 16, 19, 24]. Hu et al. conducted a meta-analysis of hip fracture among the elderly and identified that the risk factors for overall mortality were older age, male, nursing home or facility residence, poor preoperative walking capacity, poor daily activities, poor mental state, and multiple comorbidities [14].

Postoperative complication rates served as performance metrics of the health care applied to manage hip fractures. We found that hemodialysis subjects also had higher medical and surgical complication rates than those of non-hemodialysis subjects. Previous studies found that short-term readmission or reoperation rates varied from 10 % to 20 % and from 19 % to 32 % within one and three months, respectively, and the long-term reoperation rates (more than two years) ranged from 20 % to 40 % after surgery for hip fractures among elderly adults [5–13]. The majority of readmissions occurred within three months [8, 12]. Several studies also found that readmissions or reoperations were associated with higher mortality and the mortality rates after complication were 35 % to 48 % within one year [5, 6, 9–12]. We found that the one-year and five-year cumulative incidences of the first surgical complication were 14.2 % and 20.6 %, respectively, for hemodialysis subjects compared to 11.0 % and 18.3 %, respectively, for non-hemodialysis subjects using competing risk analysis. We found that once subjects had their first reoperation, 41.9 % of hemodialysis subjects died within one year compared to 21.2 % of non-hemodialysis subjects. We also found that the three-month cumulative incidence of the first medical complication was 24.1 % for hemodialysis subjects compared to 18.4 % for non-hemodialysis subjects. Once subjects had the first medical complication, 39.9 % of hemodialysis subjects died within three months compared to 19.0 % of non-hemodialysis subjects.

Osteoporotic hip fracture, aging, and end-stage renal disease all accelerate the mortality rate. Thus, it is not possible to simply estimate the cumulative complication rate without considering competing death. The interpretation of Kaplan–Meier estimates in the presence of competing risks often overestimates the risk or benefit of some covariates. This may result in over-treating risk factors with possible adverse side-effects on hip fracture subjects with hemodialysis [31, 32]. In addition, competing death rate may have different effects on short-term medical complication rates and long-term surgical complication rates. In our study, older age, trochanteric fracture, and hemiarthroplasty had significantly higher hazard ratios for mortality and short-term medical complication. However, older age, trochanteric fracture, and hemiarthroplasty had significantly lower hazard ratios for surgical complication. For example, subjects with trochanteric fracture had a higher hazard ratio for mortality (HR = 1.235) than those with cervical fracture such that subjects with cervical fracture had a longer survival time during follow-up and were thus exposed to more risk of surgical complications. When the follow-up time was short, the cumulative mortality rate was still low; many subjects with trochanteric fracture still had a higher hazard ratio for short-term medical complications (sub-HR = 1.262) than those with cervical fracture. However, when the follow-up time was longer, there were fewer subjects with trochanteric fracture during follow-up but they were healthier and therefore exposed to less risk for long-term surgical complications. Therefore, subjects with trochanteric fracture had a lower hazard ratio for long-term surgical complications (sub-HR = 0.629) than those with cervical fracture. In some subpopulations, the incidence of competing death may be much larger than the complication incidence which limits the benefit of using costly treatments to reduce certain complications. The effects of a risk factor on competing death rate and complication rate are often different and increase the difficulty of conducting the analysis and interpretation. In practice, competing risk analysis is a preferable method for directly assessing actual risk and provides more relevant results, which are valuable for prognosis and medical decision-making [31–34].

Among hemodialysis subjects, we found that 29 % and 23 % of 474 and 646 subjects with at least one medical complication within one and three months, respectively, had acute renal failure. Most research on the incidence and causes of acute renal failure after major surgery is based on studies of patients who received cardiac or vascular surgery [36–41]. Previous studies found that older age, higher BMI, diabetes, hypertension, chronic obstructive pulmonary disease, liver disease, hyperlipidemia, malnutrition, abnormal renal function, use of angiotensin-converting enzyme inhibitors (ACEIs) or angiotensin receptor blockers (ARBs), diuretics, nonsteroidal anti-inflammatory agents, infection, emergency/urgent surgery, and high-risk surgery were related to acute renal surgery after major surgery [36–41]. Various risks contributed to acute renal failure in dialysis patients after surgery for hip fracture, including comorbidities, co-medications, dialysis time before or after surgery, perioperative abnormal laboratory value, optimal time for surgery, type of anesthesia, type of operation, and intraoperative management, and these variables are likely similar to those associated with increased risk of acute renal failure following major cardiac or vascular surgery.

We also found that subjects with chronic heart disease had a higher hazard ratio for mortality and medical complication than those without chronic heart disease. Bone mineral disorders such as vitamin K deficiency, vitamin D deficiency, hypocalcemia, secondary hyperparathyroidism, hyperphosphatemia, bone loss, malnutrition, multiple metabolic mediators, and uremic toxins are present in nearly all dialysis subjects and are related to the risk of fracture risk, hypertension, vascular calcifications, and cardiovascular disease. Studies also found that vitamin K deficiency decreased bone mass and increased excessive vascular calcification. Rigorous corrections on bone mineral disorders and malnutrition, as well as vitamin D and K supplementation are necessary to prevent hip fracture in dialysis subjects [42–50].

### Limitations

Our results were based on hospitalized patients who underwent hip fractures and received operations. Selection biases may therefore have existed. Certain unknown confounding factors might have occurred or changed during the follow-up period. Although we used a matched cohort design and conducted a multivariable analysis to examine risk factors, many risk factors, such as pre-operative joint function/condition, renal function, smoking status, body mass index, bone mineral density, lifestyle, comorbidity severity, and quality of life, were not available for adjustment. The 90-day medical complication and short-term complication rates might vary depending on the definitions used [23, 25]. Therefore, directly comparing our results to those of other studies may not be meaningful because of the different definitions of the complications used in the studies.

## Conclusions

Between 1999 and 2007 in Taiwan, the overall mortality rate and complication rate of hemodialysis subjects after surgery for hip fracture were significantly higher than those of non-hemodialysis subjects. Hemodialysis subjects had a 2.32 times higher hazard ratio of overall death, a 1.15 times higher sub-hazard ratio of surgical complications, and a 1.35 times higher sub-HR of the first medical complication than non-hemodialysis controls. Further prospective studies which include important risk factors are necessary to more precisely quantify the adjusted effect of hemodialysis.

## Consent

The NHI Research Database is composed of anonymous secondary data released to the public for research purposes. Thus, this study was exempted from a full review by the local ethics review committee.

## Additional files

Additional file 1:
**Causes of medical complications after surgery for hip fracture, stratified by hemodialysis groups.**
Additional file 2:
**Causes of surgical complications after surgery for hip fracture, stratified by hemodialysis groups.**
Additional file 3:
**Mortalities within one year after causes of first medical or first surgical complications after surgery for hip fracture, stratified by hemodialysis groups.**
