# Supplementary material for: Mortality and complications after hip fracture among elderly patients undergoing hemodialysis
Source: BMC Nephrol. 2015 Jul 7;16:100. doi: 10.1186/s12882-015-0099-0 (PMC4492013; doi:10.1186/s12882-015-0099-0)
Supplement: Additional file 2: — Causes of surgical complications after surgery for hip fracture, stratified by hemodialysis groups. [file 12882_2015_99_MOESM2_ESM.doc]

### Additional file 2. Causes of surgical complications after surgery for hip fracture, stratified by hemodialysis groups

|  | **1-Month** | | **3-Month** | | **6-Month** | | **1-year** | | **2-year** | | **5-Year** | | **10-Year** | |
| --- | --- | --- | --- | --- | --- | --- | --- | --- | --- | --- | --- | --- | --- | --- |
| **Surgical complication** | **Non-HD^a^** | **HD** | **Non-HD** | **HD** | **Non-HD** | **HD** | **Non-HD** | **HD** | **Non-HD** | **HD** | **Non-HD** | **HD** | **Non-HD** | **HD** |
| At least one complication, n^a^ | 114 | 175 | 180 | 265 | 238 | 324 | 301 | 386 | 365 | 460 | 462 | 527 | 490 | 540 |
| Convert to or revision arthroplasty^b^ | 22.81% | 7.43% | 34.44% | 20.38% | 39.08% | 26.54% | 42.19% | 31.61% | 43.84% | 34.35% | 45.45% | 35.48% | 44.69% | 35.93% |
| Infection | 31.58% | 44.00% | 29.44% | 36.98% | 26.89% | 34.57% | 24.92% | 31.61% | 22.47% | 30.87% | 21.21% | 32.26% | 21.43% | 32.04% |
| Removal of implant | 12.28% | 7.43% | 17.78% | 15.47% | 22.69% | 19.44% | 26.58% | 21.76% | 25.75% | 23.04% | 24.03% | 22.96% | 22.86% | 22.59% |
| Mechanical complication^c^ | 27.19% | 29.71% | 35.00% | 36.60% | 33.61% | 38.58% | 31.56% | 36.27% | 29.86% | 33.48% | 27.49% | 31.69% | 26.53% | 31.30% |
| Dislocation | 12.28% | 12.57% | 12.78% | 16.60% | 10.92% | 14.81% | 8.64% | 12.69% | 7.40% | 10.87% | 6.49% | 9.68% | 6.33% | 9.44% |
| Avascular necrosis | 13.16% | 8.00% | 8.33% | 5.28% | 6.30% | 4.32% | 4.98% | 3.63% | 4.11% | 3.04% | 3.25% | 2.66% | 3.06% | 2.59% |
| Same site hip fracture | 4.39% | 2.29% | 5.56% | 4.53% | 4.62% | 4.63% | 5.32% | 4.92% | 6.85% | 5.43% | 8.44% | 6.45% | 8.78% | 6.67% |
| Malunion/Nonunion | 1.75% | 0.00% | 2.22% | 2.26% | 4.62% | 4.94% | 6.98% | 6.99% | 7.40% | 8.48% | 6.28% | 7.78% | 5.92% | 7.59% |

^a^n = The number of subjects with at least one readmission or reoperation.

^b^% = Percentage of subjects who had a particular cause of complication among the total number of subjects who had at least one complication. Subjects might have more than one readmission or operation due to multiple causes.

^c^Mechanical complications included loss reduction, screw back out or cutting out, skin irritation, implant broken/failure.
