# Supplementary material for: Mortality and complications after hip fracture among elderly patients undergoing hemodialysis
Source: BMC Nephrol. 2015 Jul 7;16:100. doi: 10.1186/s12882-015-0099-0 (PMC4492013; doi:10.1186/s12882-015-0099-0)
Supplement: Additional file 3: — Mortalities within one year after causes of first medical or first surgical complications after surgery for hip fracture, stratified by hemodialysis groups. [file 12882_2015_99_MOESM3_ESM.doc]

### Additional file 3. Mortalities within one year after causes of first medical or first surgical complications after surgery for hip fracture, stratified by hemodialysis groups

|  | **One-Year Mortality** | |
| --- | --- | --- |
| **Hemodialysis Group** | **after first Medical Complication** | **after first Surgical Complication** |
| **Non-HD** | 36.6% | 21.2% |
| **HD** | 63.6% | 41.9% |
